# The yeast prefoldin-like URI-orthologue Bud27 associates with the RSC nucleosome remodeler and modulates transcription

**DOI:** 10.1093/nar/gku685

**Published:** 2014-07-31

**Authors:** María Carmen Mirón-García, Ana Isabel Garrido-Godino, Verónica Martínez-Fernández, Antonio Fernández-Pevida, Abel Cuevas-Bermúdez, Manuel Martín-Expósito, Sebastián Chávez, Jesús de la Cruz, Francisco Navarro

**Affiliations:** 1 Departamento de Biología Experimental, Facultad de Ciencias Experimentales, Universidad de Jaén, Paraje de las Lagunillas, s/n, 23071, Jaén, Spain; 2 Instituto de Biomedicina de Sevilla (IBiS), Hospital Universitario Virgen del Rocío/CSIC/Universidad de Sevilla, E-41013 Sevilla, Spain; 3 Departamento de Genética, Universidad de Sevilla, E41012 Sevilla, Spain

## Abstract

Bud27, the yeast orthologue of human URI/RMP, is a member of the prefoldin-like family of ATP-independent molecular chaperones. It has recently been shown to mediate the assembly of the three RNA polymerases in an Rpb5-dependent manner. In this work, we present evidence of Bud27 modulating RNA pol II transcription elongation. We show that Bud27 associates with RNA pol II phosphorylated forms (CTD-Ser5P and CTD-Ser2P), and that its absence affects RNA pol II occupancy of transcribed genes. We also reveal that Bud27 associates *in vivo* with the Sth1 component of the chromatin remodeling complex RSC and mediates its association with RNA pol II. Our data suggest that Bud27, in addition of contributing to Rpb5 folding within the RNA polymerases, also participates in the correct assembly of other chromatin-associated protein complexes, such as RSC, thereby modulating their activity.

## INTRODUCTION

The unconventional prefoldin Rpb5 interactor (URI/RMP), and its orthologue in yeast, Bud27, are members of the prefoldin (PFD) family of adenosine triphosphate (ATP)-independent molecular chaperones (1–4). URI was originally identified as a protein binding the Rpb5 subunit of all three nuclear RNA polymerases (RNA pol) (5) and is considered to function as a scaffold protein able to assemble additional members of the PFD family in both human and yeast (2,3,6). Notably, we and others have demonstrated that Bud27 also binds Rpb5 in the three RNA polymerases (3,7). Furthermore, URI has been linked to essential cellular processes, such as translation initiation (8), genome stability and DNA damage response, and it is required for viability in *Drosophila*, *Caenorhabditis* and human cells (2,9,10).

Both URI and Bud27 have been reported to be involved in transcription. Human URI has been described as a negative regulator of androgen receptor (AR)-mediated transcription, able to modulate AR recruitment to target genes (10). In fact, a role for human URI as a transcriptional co-repressor has been proposed, through its interaction with the general transcription factor TFIIF, which also binds Rpb5, and with the hepatitis virus X protein (HBx) (5,11,12).

URI associates with Rvb1 and Rvb2, conserved essential AAA+ ATP-dependent DNA helicases that are present in the chromatin remodeling/modifying complexes Ino80, Swr-C, BAF and Tip60 (7,13,14). Furthermore, human URI associates with the PAF1 complex, which promotes RNA pol II (CTD) phosphorylation and histone modification during transcription elongation (15).

Despite all these evidences for a role of URI in transcription in different organisms, the precise role of Bud27 in transcription has not yet been deciphered in *Saccharomyces cerevisiae*. We have previously demonstrated that Bud27 shuttles between the cytoplasm and nucleus in an Xpo1-independent manner (3). Most importantly, Bud27 associates with the RNA polymerases in the cytoplasm, mediating its cytoplasmic assembly in an Rpb5-dependent manner (3).

In this work, we present evidence that Bud27 contributes to mRNA biogenesis and RNA pol II transcription elongation. We show that the lack of Bud27 affects RNA pol II occupancy within genes. Moreover, we demonstrate that Bud27 associates with RNA pol II phosphorylated forms (CTD-Ser5P and CTD-Ser2P), and modulates transcription elongation. We also reveal that Bud27 associates *in vivo* with the Sth1 component of chromatin remodeling complex RSC and mediates its association with RNA pol II. Our data suggest that Bud27 contributes to the folding of Rpb5 within the RNA polymerase II thereby modulating its activity, and participates in the correct assembly of other chromatin-associated factors, such as the RSC complex.

## MATERIALS AND METHODS

### Yeast strains, plasmids, genetic manipulations, media and genetic analysis

The *S. cerevisiae* strains used in this study are listed in Supplementary Table S1. The media preparation, yeast transformation and genetic manipulations were performed according to established procedures described elsewhere (16). Mycophenolic acid (MPA), 6-Azauracil (6AU) and rapamycin were used at the indicated concentrations.

All recombinant DNA techniques were performed according to established procedures using *Escherichia coli* XL1-blue for cloning and plasmid propagation. Plasmids used in this study are listed in Supplementary Table S2. Oligonucleotides are listed in Supplementary Table S3.

### Two-hybrid analysis

Two-hybrid analysis were performed as described (17). In this approach, *pAS2Δ-RPB5* fusion of Rpb5 to the Gal4p DNA binding domain was used as bait and *pACT2-BUD27*(368–1285), containing a fragment of Bud27 fused to the Gal4p DNA interacting domain as prey. *pAS2Δ-rpb5x* correspond to the different *rpb5* mutant alleles analysed. *pAS2Δ* and *pACT2* were also used as negative controls.

### Protein immunoprecipitation and tandem affinity purification (TAP)

First, 400 ml of the appropriate cells growing exponentially (OD_600_ ∼ 0.6–0.8) in yeast extract-peptone-dextrose (YPD) or synthetic minimal (SD) media were washed twice with ultrapure water and lysis buffer (50 mM HEPES [pH 7.5], 120 mM NaCl, 1 mM ethylenediaminetetraacetic acid, 0.3% Chaps). Then cells were resuspended in 1 ml of lysis buffer supplemented with 1× protease inhibitor cocktail (Complete, Roche), 0.5 mM phenylmethanesulfonylfluoride (PMSF), 2 mM sodium orthovanadate and 1 mM sodium fluoride and whole-cell extracts were prepared using a MixerMill MM400 RETSCH® (3 min 30Hz). Immunoprecipitations were carried out as described elsewhere (18) with some modifications: 150 μl of whole-cell extract (2000 μg protein) per experiment and lysis buffer for all washes were used. Also, 35 μl of Dynabeads M-280 Sheep anti-Mouse IgG (Invitrogen) were mixed with 9E10 anti-C-Myc antibody (1μg, Santa Cruz Biotechnology), 8WG16 anti-Rpb1 antibody (1.5 μg, Covance) or 12CA5 anti-HA antibodies (0.4 μg, ROCHE).

For TAP purification, the same protocol was applied with Dynabeads Pan Mouse IgG (Invitrogen). The affinity-purified proteins were released from the beads by boiling for 10 min. Eluted proteins were analysed by western blot with different antibodies: 9E10 anti-C-Myc (Santacruz), 8WG16 anti-Rpb1 (Santacruz), PAP (Sigma), anti-POLR2C (anti-Rpb3; 1Y26, Abcam), anti-CTD-Ser5 (anti-RNA polymerase II; CTD4H8, Millipore) or anti-CTD-Ser2 (anti-RNA polymerase II; ab5095, Abcam).

### Chromatin isolation

Chromatin isolation was performed as previously described (19) with some modifications. Briefly, about 5 × 10^8^ cells growing exponentially (OD_600_ ∼ 0.6–0.8) were resuspended in 3 ml of 100 mM PIPES/KOH (pH 9.4) containing 10 mM DTT and 0.1% sodium azide and then incubated at room temperature for 10 min. After being spun down, cells were resuspended in 2 ml of 50 mM phosphate buffer (pH 7.5), containing 0.6 M Sorbitol, 10 mM DTT and 4 μl of 20 mg/ml zymoliase and were incubated 10 min at 37°C in a water bath to spheroplast formation. Spheroplasts were then pelleted at 4°C, were washed with 50 mM HEPES-HOK buffer (pH 7.5) containing 100 mM KCl, 2.5 mM MgCl_2_ and 0.4 M Sorbitol, were resuspended in equal volume (∼80 μl) of EBX buffer (50 mM HEPES/KOH, pH 7.5), containing 100 mM KCl, 2.5 mM MgCl_2_, 0.25% Triton-X100, 0.5 mM PMSF, 0.5 mM DTT and 1× protease inhibitor cocktail (Complete; Roche), and were incubated 3 min on ice. Spheroplasts break under these conditions and the resulting whole-cell extracts were added to 400 μl of EBX-S buffer (EBX with 30% sucrose) and centrifuged at 12 000 revolutions per minute (rpm) for 10 min. After centrifugation, a chromatin pellet was visible, which was washed with 400 μl of EBX buffer and finally resuspended in 50 μl of 1.5× Tris-Glycine SDS Sample Buffer and incubated for 2 min at 85°C, followed by spinning at 10 000 rpm for 30 s. A 1:3 dilution of chromatin pellet was used for sodium dodecyl sulphate-polyacrylamide gel electrophoresis and western blotted with anti-Histone H3 (ab1791; Abcam), anti-phosphoglycerate kinase, Pgk1, (459250; Invitrogen) or PAP (Sigma) antibodies.

### Western blots quantification

Intensities of immunoreactive bands on western blots were quantified by densitometry using the software TOTALLAB from images acquired with and office scanner. The data are the results of at least three different experiments.

### Chromatin immunoprecipitation (ChIP)

ChIPs were performed using the 8WG16 anti-Rpb1 antibody as previously described (20). For real-time polymerase chain reaction (PCR) a 1:100 dilution was used for input and a 1:4 dilution was used for immunoprecipitated samples.

Genes were analysed by quantitative real-time PCR in triplicate with at least three independent biological replicates using SYBR premix EX Taq (Takara).

Values found for the immunoprecipitated PCR products were compared to those of the total input, and the ratio of values from each PCR product of transcribed genes to the value of a non-transcribed region of chromosome V was calculated. The oligonucleotides used are listed in Supplementary Table S3.

### Extraction of mRNA and reverse transcription

Total RNA from yeast cells was prepared as described (21). First-strand cDNA was synthesized using 1 μg of RNA with iScript cDNA synthesis kit (Bio-Rad) following the manufacturer's protocol. As a negative control for genomic DNA contamination, each sample was subjected to the same reaction without reverse transcriptase.

### Quantitative real-time PCR

Real-time PCR was performed within a CFX-96 or CFX-384 Real-Time PCR instrument (BioRad) and EvaGreen detection system ‘SsoFast™ EvaGreen® Supermix’ (BioRad). Reactions were performed in 96- or 384-well plates with optical sealing tape (Bio-Rad) in 10-μl total volume containing cDNA corresponding to 0.1 ng of total RNA. Each PCR reaction was performed at least three times to have a representative average, and with three independent biological replicates. The values were normalized to levels of the 18S rRNA. The oligonucleotides used are listed in Supplementary Table S3.

### GLAM assay

The Gene Length Accumulation of mRNA (GLAM) assay was carried out as previously described (22) with cells grown to mid-log phase in selective synthetic medium lacking uracil and containing 2% galactose. Three to four independently induced cultures for each strain were assayed. The mean values and standard deviations are represented in the corresponding figures.

### Structure modelling

Atomic coordinates of the yeast RNA pol II were retrieved from the Protein Data Bank (PDB, RCSB) with the accession number PDB 1WCM, and visualized with the PyMOL program (DeLano Scientific LLC) (23).

## RESULTS

### *BUD27* genetically interacts with *RPB5*

We have previously shown that Bud27 shuttles between the nucleus and the cytoplasm and mediates the assembly of the three RNA polymerases in *S. cerevisiae* in an Rpb5-dependent manner (3). A role for the Bud27 homologue, URI, in transcription has been proposed in humans and other metazoa (5,7,24). Considering these data, we investigated whether yeast Bud27 could also have a nuclear role in transcription.

**Figure 1.**
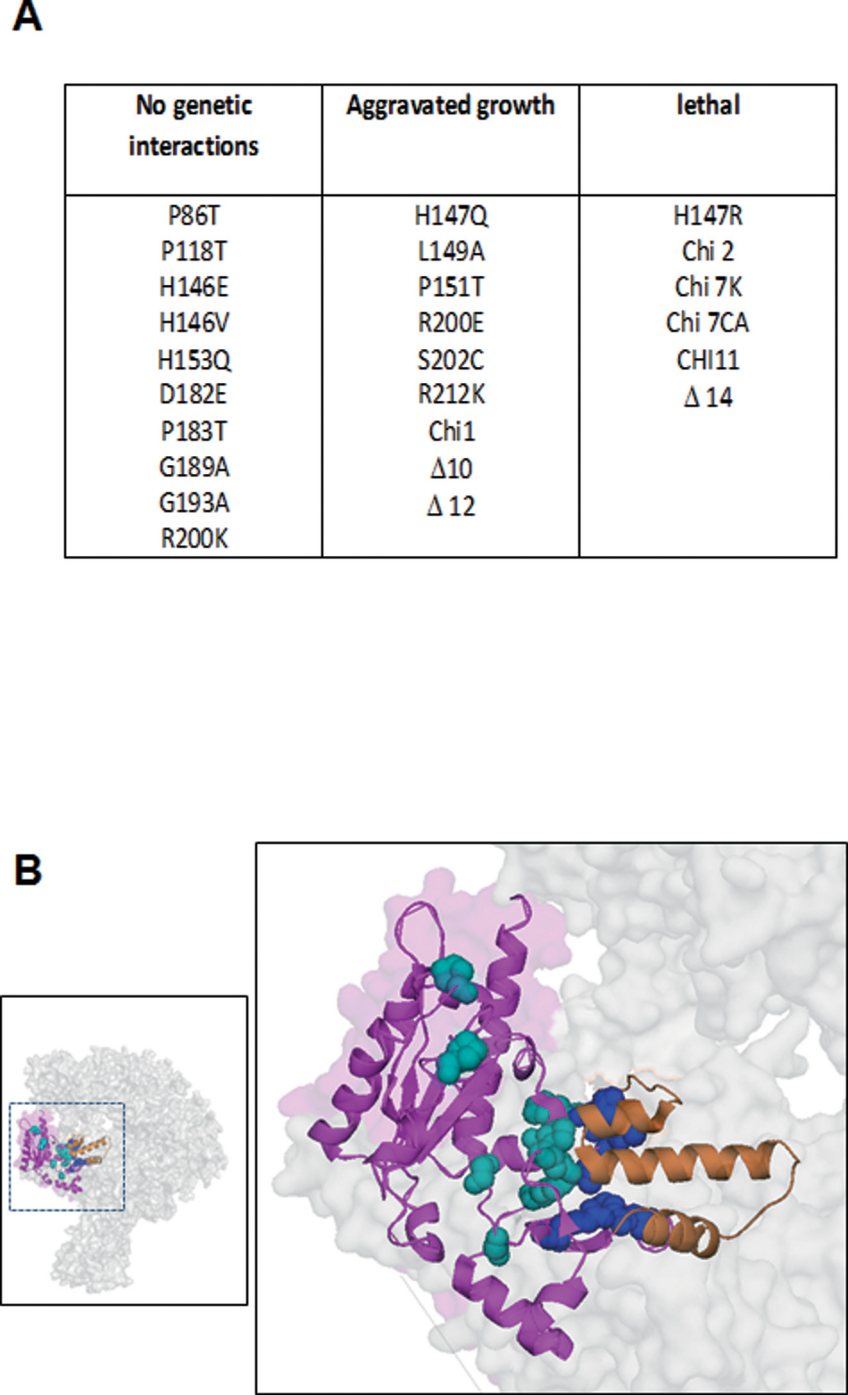
*BUD27* and *RPB5* genetically interact. (**A**) List of genetic interactions between *rpb5* and *bud27Δ* mutants based on their growth in YPD plates. (**B**) Left panel, schematic view of Rpb5 (purple) and the cleft domain (orange) of the RNA pol II of *S. cerevisiae* on the structure of the complex, and the position of Rpb5 residues whose point mutations affect (blue) or do not affect growth (cyan). Right panel, zoom view of the section indicated in the left panel.

Bud27/URI physically binds different nuclear proteins involved in transcription, among them Rpb5, a shared subunit of the three nuclear RNA polymerases (3,5,7,11,12,15). However, the genetic interaction between *BUD27* and *RPB5* in *S. cerevisiae* has never been examined. We tested for conditional synthetic interactions between 25 previously generated *rpb5* mutations (25) and the *bud27Δ* allele. As shown in Figure 1A, 15 out of the 25 double mutants were lethal or aggravated their growth with respect to the single mutants. Notably, as shown in Figure 1B, all the *rpb5* point mutations affecting growth of the *bud27Δ* strain lie at the C-terminal globe of Rpb5. This motif, which is conserved from archaea to higher eukaryotes, is involved in the interaction of Rpb5 with Rpb1, in a region directly associated with the RNA pol II cleft domain (α45–47 of Rpb1). In addition to some yeast-human chimeric *RPB5* alleles, the other *rpb5* mutations (Δ10, Δ12 and Δ14) exhibiting genetic interaction with *bud27Δ* corresponded to a different highly conserved motif (positions 11–30), belonging to the long hydrophilic helix Rpb5-α1 that occupies the ‘lower’ far-end of the DNA cleft (25,26). These results are consistent with our previous data showing that *RPB5* overexpression suppresses the thermosensitivity and rapamycin-sensitivity phenotypes of the *bud27Δ* strain (3). It bears noting that these genetic interactions reflect the participation of both proteins in common biological process and seems not to be the consequence of a loss of physical contact between Rpb5 and Bud27, as we demonstrated by two-hybrid analyses (Supplementary Figure S1).

Overexpression of other genes encoding elements of the transcriptional machinery, i.e. *RPB4*, *RPB7*, *RPB9*, *DST1* or *SPT5*, in the *bud27Δ* strain caused no effect on growth. In contrast, overexpression of *RPB6*, a common subunit to the three RNA polymerases, partially suppressed temperature sensitivity of the *bud27Δ* strain, a fact that probably accounts for the role of Bud27 in RNA pol assembly (3). Similarly, the deletion of *DST1*, a gene showing genetic interactions with *RPB5* (25), did not alter the growth of cells lacking *BUD27* (data not shown). These data reinforce the functional significance of the interaction between *BUD27* and *RPB5* in *S. cerevisiae*.

### Lack of Bud27 affects RNA pol II transcription

**Figure 2.**
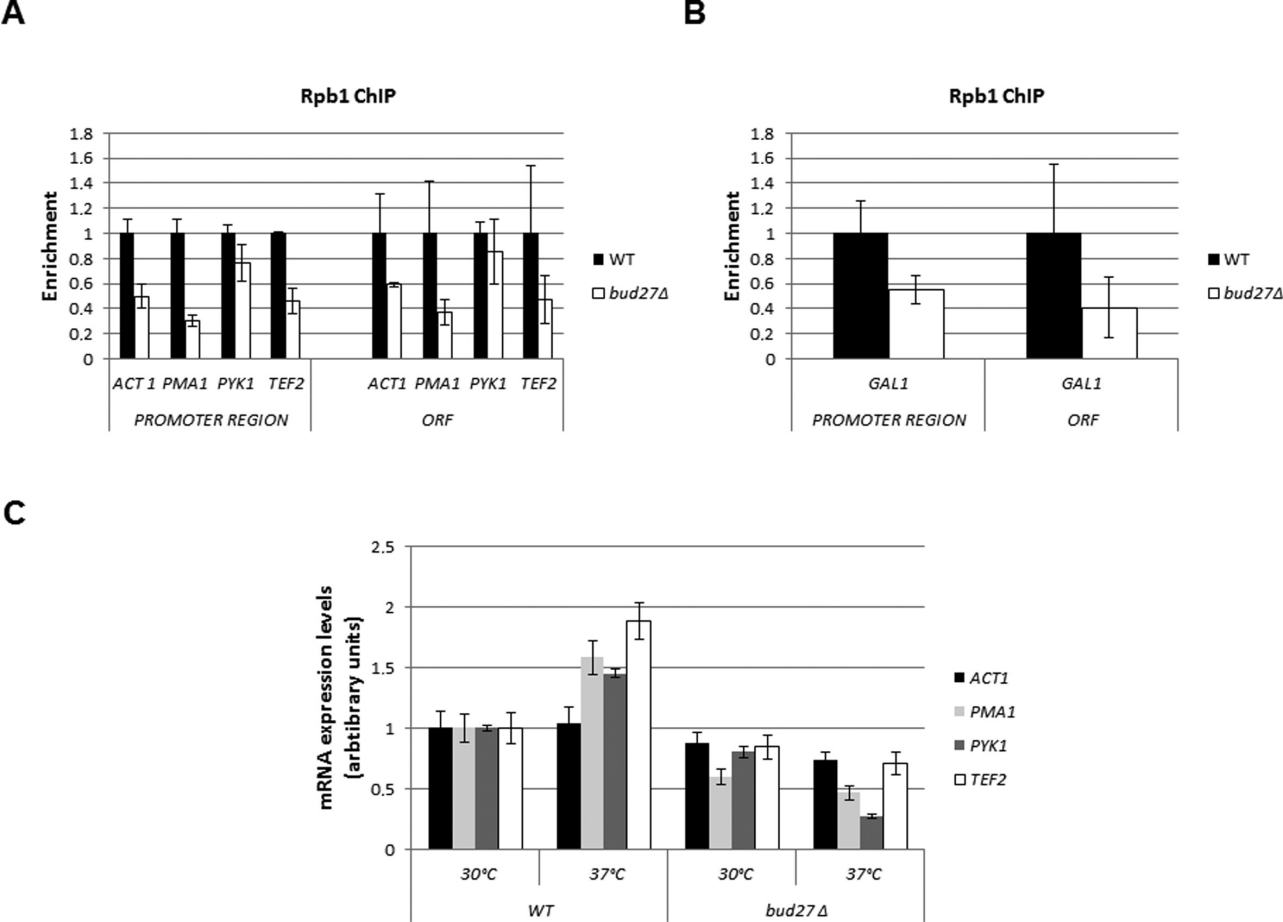
Lack of Bud27 affects RNA pol II occupancy and mRNA accumulation. (**A**) ChIP analysis for different genes in wild-type and *bud27Δ* cells, performed with 8WG16 antibodies, against the CTD repeat of the Rpb1 protein. (**B**) ChIP analysis for the *GAL1* gene under galactose-induced conditions in wild-type and *bud27Δ* cells. As above, precipitations were performed with 8WG16 antibodies. (**C**) Quantitative RT-PCR analysis of mRNA levels for different genes in wild-type and *bud27Δ* cells at 30ºC and after a 12-h shift to 37ºC. In A and B, the fold enrichment of the indicated gene ChIP samples relative to Whole Cell Extract (WCE) samples is plotted.

To investigate whether Bud27 participates in transcription, we first analysed the RNA pol II occupancy of a set of constitutively expressed genes (*ACT1*, *PMA1*, *PYK1* and *TEF2*), using ChIP experiments with the 8WG16 antibody (raised against the largest subunit of the RNA pol II, Rpb1). As shown in Figure 2A, Rpb1 occupancy decreased in *bud27* null-mutant cells both at promoters and Open Reading Frames (ORFs), although to a different extent, depending on the gene. We also analysed the RNA pol II occupancy of the inducible gene *GAL1* under activating conditions. Again, the Rpb1 ChIP signal diminished both at the promoters and the body of the gene in *bud27* null-mutant versus wild-type cells (Figure 2B). This decline in RNA pol II occupancy is consistent with, and likely the consequence of, the lower amount of enzyme transported to the nucleus under Bud27 deficiency (3).

To gain insight into the consequences of deleting *BUD27* for gene expression, we used quantitative reverse transcriptase (RT)-PCR (qRT-PCR) to compare the mRNA levels of these genes in the *bud27Δ* mutant versus the wild type at 30ºC, and after a 12-h shift to 37ºC. As shown in Figure 2C, the lack of Bud27 did not diminish mRNA levels for the genes tested at 30ºC, but impaired the accumulation of their mRNAs when shifted to 37ºC.

We conclude that *bud27Δ* exerts an impact on RNA pol II occupancy of genes and on the steady-state levels of mRNAs upon an environmental change (e.g. temperature). This is likely a transcriptional phenomenon, although we cannot rule out a Bud27-dependent effect either on mRNA stability or on RNA pol II assembly at high temperature.

### Bud27 influences RNA pol II transcription elongation and associates with phosphorylated forms of Rpb1 and chromatin

**Figure 3.**
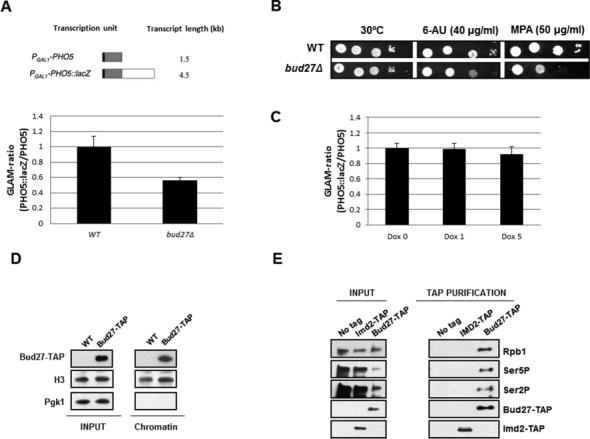
Bud27 modulates transcription elongation. (**A**) Upper panel, transcription units used in this work. Lower panel, GLAM assay in wild-type and *bud27Δ* cells. (**B**) Growth of wild-type and *bud27Δ* strains in media containing drugs MPA and 6-AZA, at 30ºC in SD medium without uracil. (**C**) GLAM assay in wild-type cells under depletion of *RPB5* by adding doxycycline at the indicated concentrations (Dox 0, Dox 1 and Dox 5 correspond to 0, 1 and 5 μg doxycycline/ml). (**D**) Chromatin fractions of a strain expressing a Bud27-TAP tagged construction from its own locus and its isogenic wild-type counterpart. Strains were grown in YPD medium at 30ºC. TAP (PAP), histone H3 and phosphoglycerate kinase (22C5D8) were analysed by western blot. (**E**) Rpb1 (8WG16), Rpb1-CTDSer5P (CTD4H8), Rpb1-CTDSer2P (ab5095), Bud27-TAP and Imd2-TAP were analysed by western in TAP purified complexes with either TAP-tagged Bud27 or Imd2 from cells growing in YPD medium at 30ºC. No tag: TAP purification with negative control extracts containing untagged Bud27.

Previously, we have proposed a role for Rpb5 in the transition from transcription initiation to elongation (25). If Bud27 influences RNA pol II-dependent transcription in an Rpb5-mediated manner, we should expect transcription elongation defects in *bud27Δ* cells. To address this question, we first investigated whether Bud27 is able to influence transcription elongation. For this, we used the length-dependent mRNA accumulation (GLAM) assay, commonly employed to detect defects in transcription elongation (22). As shown in Figure 3A, the GLAM ratio for *bud27Δ* mutant was significantly reduced compared to that observed for the wild-type strain. Consistently, we found that the growth of *bud27Δ* cells was impaired in the presence of 6-azauracil (6AU) and MPA, two Nucleotide Triphosphate (NTP)-depleting drugs used to detect *S. cerevisiae* strains defective for transcription elongation (27,28) (Figure 3B). These results strongly suggest a transcription elongation defect in the *bud27* null strain.

Taking into account that Bud27 mediates the assembly of RNA pols in an Rpb5-dependent manner and that the deletion of *BUD27* mislocalizes RNA pols in the cytoplasm (3), we considered the possibility of the GLAM ratio defect being mediated by an impaired loading of Rpb5 onto RNA pol II. To test this hypothesis, we analysed the GLAM ratios in a conditional strain that expressed *RPB5* under the control of a doxycycline repressible promoter (25,29). When we depleted Rpb5 with increasing concentrations of doxycycline, both the GLAM ratios (Figure 3C), as well as the mRNA levels of the transcription units remained practically unaffected (Supplementary Figure S2).

These results suggest that Bud27 has a role in transcription elongation which is apparently independent of the loading of Rpb5 in the RNA pol II, although our results do not rule out that this might be due to a defect in the Rpb5 folding inside the RNA pol II.

Also, we investigated Bud27 occupancy of transcribed genes by performing ChIP assays in a strain expressing a functional Bud27-TAP protein. The analysis of the 5′ ends and internal regions of different genes by qPCR ChIP assays showed that Bud27 does not significantly bind RNA pol II genes under these experimental conditions (Supplementary Figure S3A). To explore this in more detail, we also analysed the *GAL1* gene under repressed or induced conditions, showing again no significant enrichment (Supplementary Figure S3B). The fact that only a small fraction of Bud27 is detected in the nucleus (3) and the possibility that it only transiently associates with chromatin, could account for the difficulty of detecting this interaction by ChIP. Thus, we performed chromatin isolation from a strain expressing a chromosomal Bud27-TAP tagged construction from its own locus (3), and analysed the presence of Bud27 by western blot with PAP antibodies. Figure 3D shows that Bud27-TAP is clearly associated with chromatin, as was also the case for the histone H3 (nuclear control). As expected, Pgk1, the cytoplasmic control included in the experiment, was not detected in association with chromatin.

Additionally, we explored the association of Bud27 to the phosphorylated forms of Rpb1 (CTD Ser5P and CTD Ser2P), which are specific markers of elongating RNA pol II (30). As shown in Figure 3E, Bud27-TAP co-immunoprecipitated with both CTD Ser5P and CTD Ser2P phosphorylated forms of Rpb1. Notably, this association is specific, as shown by using an Imd2-TAP tagged strain as a negative control.

**Figure 4.**
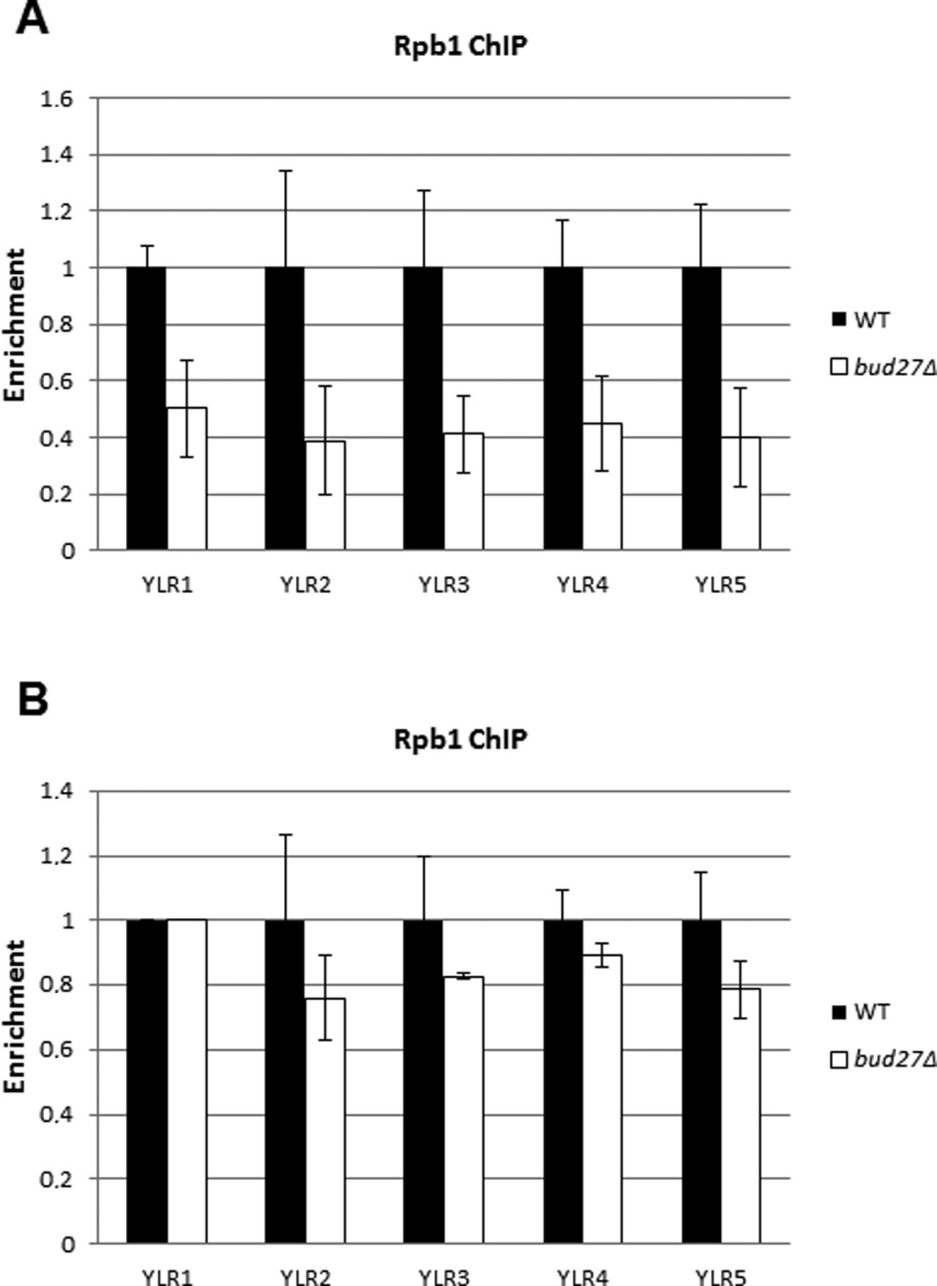
Lack of Bud27 does not significantly affect RNA pol II occupancy along the *GYLR154* unit. (**A** and **B**) ChIP analysis for *GYLR454* unit in wild-type and *bud27Δ* cells, grown in in SD medium containing galactose as carbon sources, performed with 8WG16 (anti-Rpb1). The fold enrichment of the indicated gene ChIP samples relative to WCE samples is plotted.

Finally, to further investigate the contribution of Bud27 in transcription elongation, we next analysed, in a *bud27Δ* mutant strain, the association of the Rpb1 subunit of the RNA pol II with the long transcription unit of the *FMP27/YLR454w* gene, expressed under the control of *GAL1* promoter (31) in a *bud27Δ* mutant strain. Similarly to what occurs with the previously constitutive genes tested (see Figure 2A), RNA pol II association decreased through the entire ORF of *FMP27/YLR454w* (Figure 4A). However, we found no significant difference in the profile of the RNA pol II occupancy in the gene when values were normalized to the 5′ amplicon (Figure 4B). This suggests that Bud27 does not influence RNA polymerase II processivity, analysed as the ability of elongating RNA pol II to travel the entire length of the gene (31).

All together, these data indicate that Bud27 is able to interact physically with the RNA pol II elongating complex, supporting a direct role for Bud27 in transcription that would account for the transcriptional defects of *bud27Δ*.

### Bud27 interacts with the chromatin remodeler RSC complex

**Figure 5.**
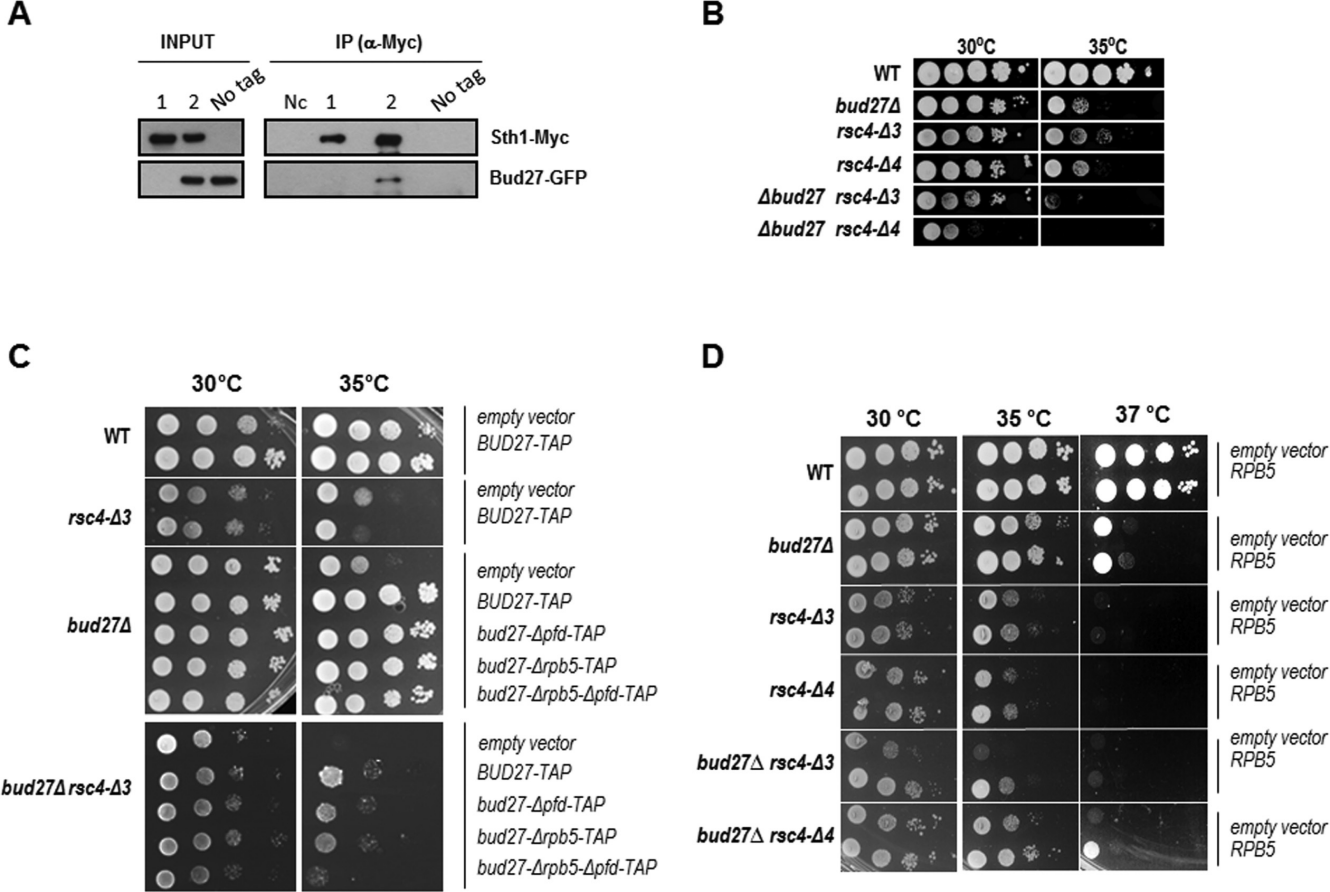
Bud27 associates with the RSC chromatin remodeling complex. (**A**) Sth1-Myc and Bud27-GFP co-immunoprecipitate. Sth1 was immunoprecipitated with anti-Myc antibodies and precipitates were analysed by western blot with anti-Myc and anti-GFP antibodies. 1 and 2, *bud27Δ* Sth1-Myc cells containing an empty vector (1) or a vector expressing Bud27-GFP (2); No tag, untagged Sth1 cells expressing Bud27-GFP from a plasmid; Nc, negative control without antibodies. (**B**) Growth of *bud27Δ*, *rsc4Δ-3* and *rsc4Δ-4* single and double mutants in YPD medium at the indicated temperatures. (**C**) Growth of wild-type and *bud27Δ*, *rsc4Δ-3* and *bud27Δrsc4Δ-3* mutants transformed with an empty vector or different *BUD27* constructions in SD medium at the indicated temperatures. (**D**) Growth of *bud27Δ*, *rsc4Δ-3* and *rsc4Δ-4* single and double mutants transformed with an empty vector or with a vector overexpressing *RPB5*, in SD medium, at the indicated temperatures.

It has been reported that Rpb5 physically interacts with the Rsc4 subunit of RSC complex, an essential multisubunit chromatin remodeling complex that participates in transcription elongation (32,33). Moreover, certain thermosensitive *rpb5* alleles are lethal in combination with a specific *rsc4* mutation (*rsc4-Δ4*), supporting the physiological significance of the Rpb5-Rsc4 interaction (18). Genetic interactions between *BUD27* and *RSC1* and *RSC8*, encoding other components of RSC, has been reported (34). In addition, Bud27 has been shown to genetically or physically associate with other elements of the yeast chromatin machinery, such as Paf1, SAGA, histone deacetylases or SWR1, among others (34–37). Based on these findings, we investigated a possible physical interaction between Bud27 and the RSC complex. We first analysed the association between Bud27 and RSC complex by performing protein co-immunoprecipitation. To do so, we introduced a functional Myc-tagged version of the Sth1 subunit of the RSC complex (18), in a b*ud27Δ* strain. We also expressed in this strain a functional Bud27-GFP protein from a plasmid (3). The *bud27Δ* strain transformed with a vector expressing Bud27-GFP was used as untagged control. As shown in Figure 5A, when Sth1-Myc was immunoprecipitated in the strain expressing Bud27-GFP (Green Fluorescent Protein), an anti-GFP reacting band was detected (line 2). In clear contrast, no such band was observed when the immunoprecipitation was performed in the control strain expressing Bud27-GFP in an untagged Sht1 background (No tag). In addition, no anti-Sth1-Myc or anti-Bud27-GFP-reacting material was adsorbed non-specifically onto the beads (Nc). These observations indicate that Bud27 specifically interacts with the Sth1 subunit of RSC complex, and probably therefore with RSC.

To evaluate the functional relevance of this interaction, we looked for synthetic phenotypes between *bud27Δ* and two *rsc4* thermosensitive alleles, *rsc4-Δ3* and *rsc4-Δ4*, deleted for the three or four last amino acids of the C-terminus of Rsc4, respectively (18). As shown in Figure 5B, a clear genetic interaction was found between *bud27*Δ and *rsc4-Δ3* and *rsc4-Δ4* alleles. In the latter case, the interaction was detected even under the permissive condition.

Uri/Bud27 is believed to function as a scaffold protein able to assemble additional members of the chaperone PFD family through its PFD and Rpb5-binding domains in both human and yeast (8). Then, to test which conserved domains of Bud27 are important for *BUD27* and *RSC4* genetic interaction, we transformed *bud27Δ rsc4-Δ3* double mutant, as well as *bud27Δ* cells with plasmids expressing full-length or deleted versions of Bud27, and tested their ability to complement the ts phenotype of the double mutant. As shown in Figure 5C, constructions lacking both the PFD and the Rpb5-binding domain could complement growth of a *bud27Δ* null strain, as previously indicated (3,8), indicating that these domains are not functionally relevant in this context. In addition, deletion of the PFD domain had a reproducibly slight impact on growth of the *bud27Δ rsc4-Δ3* double mutant, suggesting that PFD domain could be involved in the functional interaction between *BUD27* and *RSC4*.

Based on the physical and genetic interaction between Bud27 and Rpb5, as well as between Rpb5 and Rsc4, and on the fact that the interaction between Rpb5 and Rsc4 C-terminus was direct and required its last four amino acids (18), we checked whether the genetic interaction between *BUD27* and *RSC4* was indeed dependent of Rpb5. To do so, we analysed whether *RPB5* overexpression suppressed the ts defect of the *bud27Δ rsc4-Δ3* and *bud27Δ rsc4-Δ4* double mutants. As shown in Figure 5D, *RPB5* overexpression partially suppressed the growth defect of a *bud27Δ* null strain, as previously reported (3), but not the growth defect of *rsc4-Δ3* and *Δ rsc4-Δ4* mutants. Moreover, overexpression of *RPB5* partially suppressed the slow-growth phenotype of the double mutants *bud27Δ rsc4-Δ3* and *bud27Δ rsc4-Δ4*, especially at 35ºC (Figure 5D).

Taken together, our data demonstrate that Bud27 physically and functionally associates with the chromatin remodeling complex RSC, an association that could partially depend on the Bud27 PFD domain. In addition, the effect of Rpb5 as a suppressor of the genetic interaction between *BUD27* and *RSC4* suggest that the interaction between Bud27 and Rpb5 could be relevant for the effect of Bud27 on the RSC complex.

### Bud27 modulates the association between Sth1 and the RNA pol II

To explore how Bud27 modulates the function of the RSC complex, we investigated whether the *bud27Δ* mutation affects the association of Sth1 to RNA Pol II.

**Figure 6.**
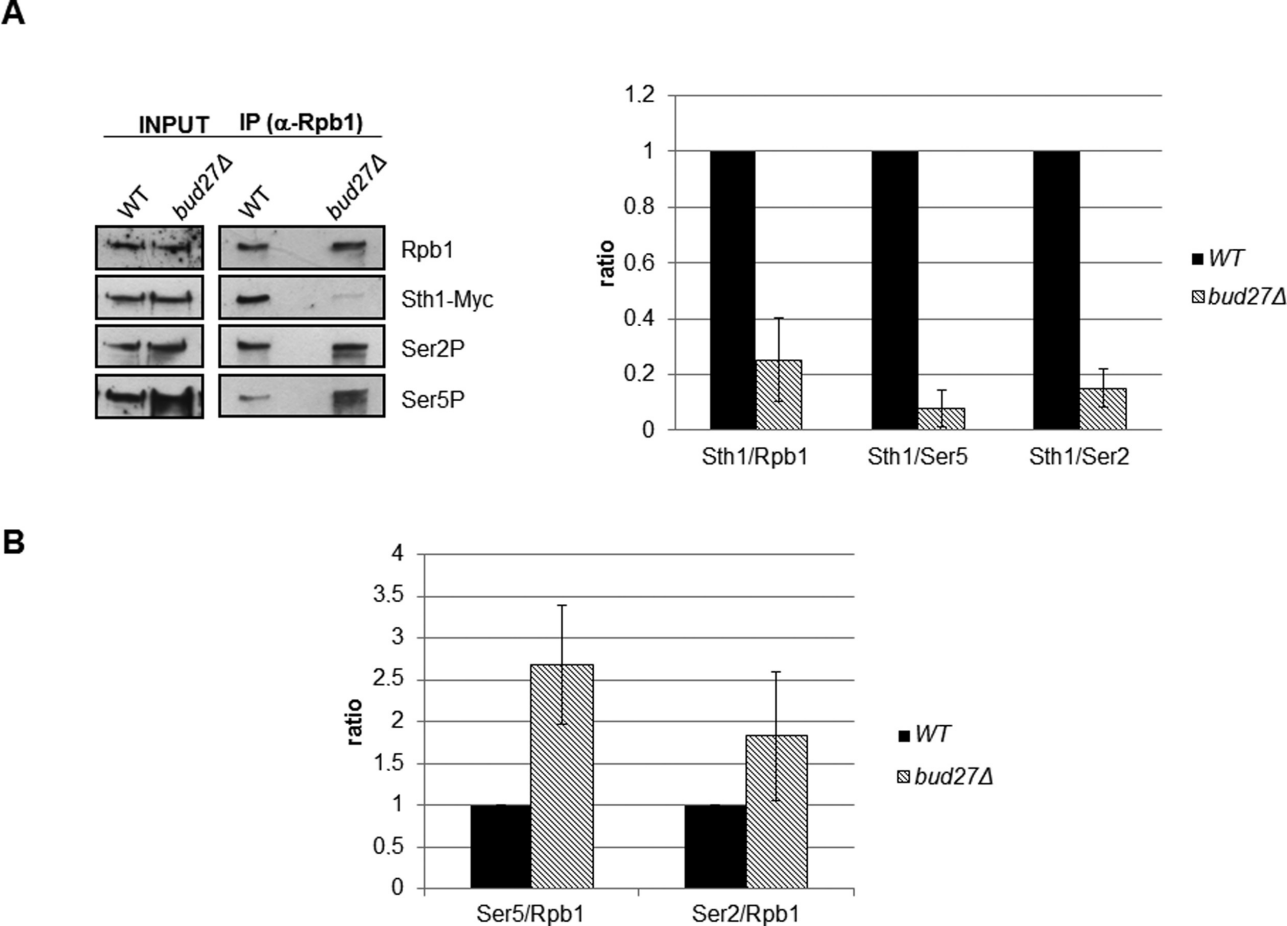
Lack of Bud27 affects the association between the RNA pol II and RSC chromatin remodeling complex. (**A**) The RNA pol II was immunoprecipitated with anti-Rpb1 antibody (8WG16), and Rpb1, Rpb1-CTDSer5P, Rpb1-CTDSer2P and Sth1-Myc were analysed by western blot with the antibodies indicated above in Sth1-Myc containing strains growing at 30ºC (left panel) in YPD medium. Ratios between Sth1 and Rpb1, Rpb1-CTDSer5P (Ser5) and Rpb1-CTDSer2P (Ser2) were calculated from the experiment in left panel (right panel). (**B**) Ratios between Rpb1-CTDSer5P (Ser5) or between Rpb1-CTDSer2P (Ser2) and Rpb1 were calculated from the experiment in A (left panel). Intensities of immunoreactive bands on western blots were quantified by densitometry using the software TOTALLAB from images acquired with office scanner. The data are the results of at least three different experiments.

Based on previously reported data showing a preferential association of RSC with promoter regions versus ORF sequences and the difficulty of analysing its association with ORFs (18,38), we investigated the association between the Sth1 subunit of the RSC complex and RNA Pol II by performing protein co-immunoprecipitation (Figure 6). For this, we used both a wild-type and a *bud27Δ* mutant strain that expressed a functional Myc-tagged version of Sth1. To immunoprecipitate RNA pol II, we used 8WG16 antibodies raised against the CTD repeats of Rpb1, the largest subunit of this RNA polymerase. We found a very significant decrease in the amount of Sth1-Myc that co-immunoprecipitated with Rpb1. In addition, as the deletion of *BUD27* leads to Rpb1 cytoplasmic accumulation (3), we analysed the levels of two phosphorylated forms of RNA pol II (CTD Ser5P and CTD Ser2P), accounting for elongating RNA pol II, in the immunoprecipitated preparations. Notably, these levels were not lower in *bud27Δ* than in the wild-type strain, and the ratios Sth1-Myc:CTD Ser2P and Sth1-Myc:CTD Ser5P in the *bud27Δ* mutant were as low as the Sth1-Myc:Rpb1 values (Figure 6A). These results were corroborated by performing similar analyses in chromatin fractions of the same strains (Supplementary Figure S4A). In addition, a sharp increase, at least in the Ser5P:Rpb1 ratio was found in *bud27Δ* with respect to a wild-type strain (Figure 6B), supporting a role by Bud27 during early elongation. Again, these data were corroborated in chromatin fractions (Supplementary Figure S4B). We conclude that Bud27 is necessary to maintain a correct association of RSC to RNA pol II during transcription. Overall, our results show a functional contribution of Bud27 to RNA pol II-dependent transcription that may be mediated by its role in promoting the interaction between the RSC complex and Rpb5, in the context of the elongating RNA polymerase.

## DISCUSSION

Bud27 is the yeast orthologue of human URI/RMP, a member of the PFD-like family of ATP-independent molecular chaperones, also called unconventional PFD Rpb5 interactor (7). Bud27 has recently been shown to mediate the assembly of the three RNA polymerases in an Rpb5-dependent manner (3), and URI has been shown to be a component of the human HSP90/R2TP complex proposed to participate in the biogenesis of RNA polymerases (1,39,40). Despite that URI coordinates interactions with elements of transcriptional machinery in humans (5,10,12,41) and affects transcription in *Drosophila* (24), the role of Bud27 in transcription has not been analysed before *in S. cerevisiae*, nor has the molecular mechanism by which this protein influences transcription. In this work, we extend the previous observation of Bud27 affecting the accumulation of some specific RNAs (7), and we present evidence that Bud27 modulates yeast transcription and influences the expression of genes transcribed by the RNA pol II.

Our data indicate that the main role for Bud27 in RNA pol II-dependent transcription would be linked to the elongation phase, since *BUD27* deletion renders cells sensitive to NTP-depleting drugs and affects length-dependent mRNA accumulation as measured by the GLAM assay (22). In addition, Bud27 associates with the elongating forms of RNA pol II, characterized by the phosphorylation of the Ser5 and Ser2 residues of its CTD domain.

One of us has recently found a role of yeast canonical PFD complex in transcription elongation (42). This finding might lead us to hypothesize that Bud27 works together with this canonical PFD complex in a concerted manner during transcription elongation. Although our data cannot absolutely exclude this possibility, several pieces of evidence indicate that Bud27 and PFD participate in transcription elongation as two distinct functional and physical entities. First, the networks of genetic interaction of PFD and Bud27 with the set of transcription elongation-related factors sharply differ. For example, the *bud27Δ* allele has no interaction with the deletion of *DST1*, the gene encoding the RNA cleavage factor TFIIS, which acts on backtracked RNA polymerase II (not shown), whereas the mutants lacking canonical PFD subunit exhibit a strong synthetic interaction in growth and in all transcriptional phenotypes tested (42). Inversely, mutations affecting the ubiquitination of H2B during transcription are synthetic with the *bud27Δ* mutation (see the Data Repository of Yeast Genetic Interaction) but are epistatic on PFD mutants (42). Secondly, the transcriptional effects of deleting *BUD27* and PFD genes differ; for instance, the deletion of PFD genes leads to the accumulation of RNA pol II towards the 3′ end of transcribed genes (42), whereas in the *bud27Δ* mutant we found lower levels of 3′:5′ RNA pol II ratios (Figure 4B). This agrees with an increase in Ser5 CTD phosphorylation (Figure 6B and Supplementary Figure S4B) and suggests a defect in the transition from initiation to elongation (43,44). Thirdly, the recruitment of Bud27 and PFD to the transcribed region is different: whereas in the first case we detected a clear co-immunoprecipitation with the elongating forms of RNA pol II (Figure 3E) but we were unable to detect a consistent ChIP signal in the transcribed region (Supplementary Figure S3), the PFD complex shows a clearly binding profile to the coding region of transcribed genes but does not co-immunoprecipitate with the Ser2-phosphorylated form of RNA pol II (42). Finally, proteomics studies of Bud27 reveal that it copurifies only with the PFD subunit Pfd6/Gim1 (3) and, in the case of human URI, with the human orthologue of Pfd2/Gim4 (40). By contrast, the transcription-linked form of PFD contains Pfd1, Pfd4/Gim3, Pfd5/Gim5 and Pfd6/Gim1, lacking Pfd2/Gim4 and Pfd3/Gim2 (42). Finally, the complex purified with a TAP-tagged version of Pfd1 apparently does not contain Bud27 (Millán-Zambrano and Chávez, unpublished). All together, these data lead us to propose that Bud27/URI and PFD plays different roles in transcription.

By interacting with elongating RNA polymerases, Bud27 would influence the recruitment of other components of the transcriptional machinery to the transcription site. In this work, we provide evidence for the role of Bud27 in the association of the chromatin remodeler RSC complex with the elongating forms of RNA pol II. We show that Bud27 binds the RSC complex. Consistently, the loss of Bud27 alters the association between Sth1 (a subunit of the RSC complex) and the elongating forms of RNA pol II. We cannot rule out that Bud27 does not mediate the association between RSC and Rpb5, since the contact between the Rsc4 and Rpb5 did not affect RSC recruitment at Pol II and Pol III genes but rather altered the chromatin structure in the promoter region of RSC-regulated genes (18). Although we clearly need to decipher the details of this triple Bud27/Rpb5/RSC interaction, we envisage a model in which Bud27 would facilitate the correct folding of both Rpb5 and its RSC partners in order to stabilize a functional interaction with the elongating RNA pol II.

Our finding that Bud27 and the RSC complex functionally interact illustrates the role of Bud27 in the transcription elongation phase. This example is consistent with published data showing that Bud27 genetically interacts with several factors involved in chromatin transaction during transcription elongation (34–37). Human URI associates with the PAF1 complex which promotes RNA pol II CTD phosphorylation and histone modification during transcription elongation (15). URI is also associated with the tumour suppressor parafibromin, suggested to be linked to mRNA processing during transcription elongation (15). Furthermore, Paf1 genetically interacts with the histone chaperone Rtt106 suggested to link transcription elongation and the chromatin dynamics associated with RNA polymerase II passage in yeast (45). In addition, human URI has been described as a regulator of the transcriptional function of the AR, which associates with the phosphorylated forms of RNA polymerase II that are indicative of the elongation phase (10). This association also agrees with those observed in humans and *C. elegans* between URI and the pontin/Rvb1-Rvb2 chromatin factor (13,14).

The data shown in this work, together with the role of Bud27/URI in the assembly of RNA polymerases (1,39,40), point to a role of Bud27 contributing to the correct folding of Rpb5 in the context of the RNA pol II, and, therefore, modulating its activity. We cannot rule out a similar role for the other two nuclear RNA polymerases. In fact, our data suggest that *BUD27* deletion affects processing of the RNA precursors synthesized by the RNA pol I and RNA pol III (not shown). Furthermore, since Rpb5 is present in all three nuclear RNA polymerases, it is tempting to speculate that Bud27 might also facilitate the described interactions of the RSC complex with RNA pol I and III (18). In this way, Bud27 action on Rpb5 might function as a global switch for genome transcription by affecting the interaction of all three RNA polymerases with factors required during transcription elongation. The well-known functional connection between the target of rapamycin (TOR) signalling pathway and Bud27/URI (7) makes this a very attractive model.

In conclusion, the transcriptional role of Bud27 that we describe here supports the importance of protein folding in transcription regulation, an emerging aspect in the gene expression field (42,46).

## SUPPLEMENTARY DATA

Supplementary Data are available at NAR Online.

SUPPLEMENTARY DATA
