# Supplementary material for: The yeast prefoldin-like URI-orthologue Bud27 associates with the RSC nucleosome remodeler and modulates transcription
Source: Nucleic Acids Res. 2014 Jul 31;42(15):9666–76. doi: 10.1093/nar/gku685 (PMC4150788; doi:10.1093/nar/gku685)
Supplement: SUPPLEMENTARY DATA [file supp_42_15_9666__index.html]

The yeast prefoldin-like URI-orthologue Bud27 associates with the RSC nucleosome remodeler and modulates transcription — SUPPLEMENTARY DATA 

# The yeast prefoldin-like URI-orthologue Bud27 associates with the RSC nucleosome remodeler and modulates transcription

## SUPPLEMENTARY DATA

**Files in this Data Supplement:**

- SUPPLEMENTARY DATA
