# Supplementary material for: The yeast prefoldin-like URI-orthologue Bud27 associates with the RSC nucleosome remodeler and modulates transcription
Source: Nucleic Acids Res. 2014 Jul 31;42(15):9666–76. doi: 10.1093/nar/gku685 (PMC4150788; doi:10.1093/nar/gku685)
Supplement: SUPPLEMENTARY DATA [file supp_gku685_nar-03668-a-2013-File008.zip › Figure captions _Tables S1-S3 JULIO2014.docx]

**Figure S1: *RPB5* mutations do not affect Rpb5-Bud27 physical interaction.** Two-hybrid analysis of wild-type *RPB5* or different *rpb5* mutant alleles (*pAS2Δ-rpb5x*) in combination with a *BUD27* construction *pACT2-BUD27(368-1285)*.

**Figure S2: *RPB5* depletion does not significantly affect the *PHO5::lacZ/PHO5* ratio.** Quantitative RT-PCR analysis of mRNA levels for *PHO5*, *PHO5::lacZ* and *RPB5* genes, in wild-type cells under *RPB5* depletion by adding doxycycline at the indicated concentrations (Dox 0, Dox 1 and Dox 5 correspond to 0, 1 and 5 μg doxycycline /ml)..

**Figure S3: Bud27 occupancy on genes. A)** Analysis of Bud27 occupancy, by chromatin immunoprecipitation, for different pol II genes in cells containing a tagged version of Bud27 (Bud27-TAP), grown in SD medium. **B)** Analysis of Bud27 occupancy for the *GAL1* gene, in cells containing a tagged version of Bud27 (Bud27-TAP), grown in SD medium containing glucose or galactose as carbon sources. The fold-enrichment of the indicated gene ChIP samples relative to WCE samples is plotted.

**Figure S4: Lack of Bud27 affects the association between the RNA pol II and chromatin remodeler RSC complex. A)** Ratios between Sth1 and Rpb1, Rpb1-CTDSer5P (Ser5) and Rpb1-CTDSer2P (Ser2) from Western blots with the antibodies against Rpb1, Rpb1-CTDSer5P, Rpb1-CTDSer2P and Sth1-Myc in chromatin fractions of Sth1-Myc containing strains growing at 30ºC in YPD medium. **B)** Ratios between Rpb1-CTDSer5P (Ser5) or between Rpb1-CTDSer2P (Ser2) and Rpb1 from experiment in A. Intensities of immunoreactive bands on Western blots were quantified by densitometry using the software TOTALLAB from images acquired with office scanner. The data are the results of at least three different experiments.

Table S1. *S. cerevisiae* strains

| **Strain** | **Genotype** | **origin** |
| --- | --- | --- |
| BY4741 | *MATa his3-Δ1 leu2-Δ0 met15-Δ0 ura3-Δ0* | Euroscarf |
| BY4742 | *MATα his3-Δ1 leu2-Δ0 lys2-Δ0 ura3-Δ0* | Euroscarf |
| D334-1a | *MAT α ade2-1 his3-Δ200 leu2-Δ1 lys2-801 trp1-Δ63 ura3-52 ppr2-Δ::hisG-URA3-hisG* | ([1](#_ENREF_1)) |
| D610-11A | *MAT α his3-Δ1 leu2-3,112 lys2-801 met15-Δ0 trp1-Δ63 ura3-1 bud27-Δ::kanMX4 rpb5-Δ::ura3::LEU2 // pFL44-RPB5* | ([2](#_ENREF_2)) |
| YFN2 | *MAT a ade2-101 his3-Δ200 leu2-Δ1 lys2-801 trp1-Δ63 ura3-52 rpb5-Δ::ura3::LEU2 // pFL44L-RPB5* | ([3](#_ENREF_3)) |
| YFN149 | *MAT α ade2-1 his3- Δ1 leu2-Δ1 met15-Δ0 trp1-Δ63 ura3-1 ppr2-Δ::hisG-URA3-hisG* | This work. D334-1a x BY4741 |
| YFN266 | *MAT α* *ade2-1 his3-Δ1 leu2-Δ1 met15Δ0 trp1-Δ63 ura3-Δ0 ppr2-Δ::hisG-URA3-hisG bud27::kanMX4* | This work. YFN149 x YFN105 |
| GYLR-4B | *MAT α his3-Δ1 leu2-Δ0 met15-Δ0 ura3-Δ0 lys2Δ0 trp1::HISG URA3::P_GAL1_-YLR454* | Gift from F. Malagón |
| MW3922 | *MAT α ade2-101 his3-∆200 leu2-∆1 lys2-801 trp1-∆63 ura3-52 rsc4-∆3::HIS3* | ([4](#_ENREF_4)) |
| MW3993 | *MAT α ade2-101 his3-∆200 leu2-∆1 lys2-801 trp1-∆63 ura3-52 rsc4-∆4::HIS3* | ([4](#_ENREF_4)) |
| Y187 | *MAT α ade2-101 his3-Δ200 leu2-3, 112 met trp1-901 ura3-52 gal4-Δ gal80-Δ URA3::GAL1_UAS_-GAL1_TATA_::lacZ* | ([5](#_ENREF_5)) |
| Y190 | *MAT a ade2-101 his3-Δ200 leu2-3, 112 lys2-801 trp1-901 ura3-52 gal4-Δ gal80-Δ URA3::GAL1_UAS_-GAL1_TATA_-lacZ LYS2::GAL4_UAS_-HIS3_TATA_-HIS3 cyh^r^2* | ([5](#_ENREF_5)) |
| YSC1178  (*BUD27-TAP*) | *MATa his3-Δ1 leu2-Δ0 met15-Δ0 ura3-Δ0 BUD27::TAP::HIS3MX6* | Open Biosystems |
| YVV9 | *MAT α ade2-1 his3-Δ200 leu2-Δ1 lys2-Δ201 trp1-Δ63 ura3-52 rpb9-Δ::HIS3* | ([6](#_ENREF_6)) |
| YFN2 | *MAT a ade2-101 his3-Δ200 leu2-Δ1 lys2-801 trp1-Δ63 ura3-52 rpb5-Δ::ura3::LEU2 // pFL44-RPB5* | ([3](#_ENREF_3)) |
| YFN13 | *MAT a ade2-101 his3-Δ200 leu2-Δ1 lys2-801 trp1-Δ63 ura3-52 rpb5-Δ::ura3::LEU2 // pCM185-RPB5* | ([3](#_ENREF_3)) |
| YFN105 | *MAT a his3Δ1 leu2Δ0 met15Δ0 ura3Δ0 trp1-Δ63 bud27::kanMX4* | ([2](#_ENREF_2)) |
| YFN106 | *MAT α his3Δ1 leu2-3,112 lys2-801 met15Δ0 ura3Δ0 trp1-Δ63 bud27::kanMX4* | ([2](#_ENREF_2)) |
| YFN222 | *MAT α his3- Δ1 leu2-Δ0 lys2-Δ0 met15-Δ0 trp1-Δ63 ura3-Δ0 URA3::P_GAL1_-YLR454 bud27::kanMX4* | This work. GYLR-4B x YFN105 |
| YFN283 | *MAT a ade2-101 his3-Δ200 leu2-Δ0 lys2-801 met15Δ0 trp1-Δ63 ura3-Δ0 rsc4-∆3::HIS3 bud27::kanMX4* | This work.  YFN105 x MW3922 |
| YFN286 | *MAT α ade2-101 his3-Δ200 leu2-Δ0 lys2-801 trp1-Δ63 ura3-Δ0 rsc4-∆4::HIS3 bud27::kanMX4* | This work.  YFN105 x MW3993 |
| YFN334 | *MATa his3Δ1 leu2Δ0 met15Δ0 trp1-Δ63 ura3Δ0 bud27::kanMX4 RPB8::ECFP::SpHIS5* | ([2](#_ENREF_2)) |
| YFN335 | *MATa his3Δ1 leu2Δ0 met15 Δ 0 ura3Δ0 lys2Δ RPB8::ECFP::SpHIS5* | ([2](#_ENREF_2)) |
| YFN359 | *MATa ura3-1 his3 Δ200 ade2-101 trp1-Δ63 leu2Δ0 lys2-801 met15Δ0 STH1::13MYC::Kan-MX6 Bud27::KanMX4* | This work. |
| YFN360 | *ura3-1 his3 Δ200 ade2-101 trp1-Δ63 leu2Δ1 met15Δ0 STH1::13MYC::Kan-MX6* | This work. |
| SCOC1836 | *MATa His3Δ1 Leu2Δ0 met15Δ0 ura3Δ0 IMD2::TAP::HIS3MX6* | Open Biosystems |

**Table S2. Plasmids**

| **Name** | **ORI and Yeast markers** | **Origin** |
| --- | --- | --- |
| *pACT2* | ORI (2µm) *LEU2* | ([7](#_ENREF_7)) |
| *pACT2-BUD27(368-1285)* | ORI (2µm) *LEU2* | ([7](#_ENREF_7)) |
| *pAS2∆* | ORI (2µm) *TRP1* | ([7](#_ENREF_7)) |
| *pAS2∆-RPB5* | ORI (2µm) *TRP1* | ([8](#_ENREF_8)) |
| *pAS2∆-rpb5x* | ORI (2µm) *TRP1* | ([8](#_ENREF_8)) |
| *pCM185* | ORI (CEN) *TRP1* | ([9](#_ENREF_9)) |
| *pCM185-RPB6* | ORI (CEN) *TRP1* | This work. |
| *pCM189* | ORI (CEN) *URA3* | ([9](#_ENREF_9)) |
| *pCM189-BUD27* | ORI (CEN) *URA3* | ([2](#_ENREF_2)) |
| *pCM189-BUD27-GFP* | ORI (CEN) *URA3* | ([2](#_ENREF_2)) |
| *pCM189-BUD27-TAP* | ORI (CEN) *URA3* | ([2](#_ENREF_2)) |
| *pCM189-BUD27ΔNES -GFP* | ORI (CEN) *URA3* | ([2](#_ENREF_2)) |
| *pCM189-BUD27ΔPFΔR5-TAP* | ORI (CEN) *URA3* | ([2](#_ENREF_2)) |
| *pCM189-BUD27ΔPFD-TAP* | ORI (CEN) *URA3* | ([2](#_ENREF_2)) |
| *pCM189-BUD27ΔR5-TAP* | ORI (CEN) *URA3* | ([2](#_ENREF_2)) |
| *pCM189-SPT15* | ORI (CEN) *URA3* | This work. |
| *pCM190* | ORI (2µm) *URA3* | ([9](#_ENREF_9)) |
| *pCM190-RPB4* | ORI (2µm) *URA3* | This work. |
| *pCM190-SPT15* | ORI (2µm) *URA3* | This work. |
| *pFL44L* | ORI (2µm) *URA3* | ([10](#_ENREF_10)) |
| *pFL44L-RPB5* | ORI (2µm) *URA3* | ([11](#_ENREF_11)) |
| *pGEN* | ORI (2µm) *TRP1* | ([3](#_ENREF_3)) |
| *pVV80* | ORI (2µm) *TRP1* | ([12](#_ENREF_12)) |
| *pGEN-RPB5* | ORI (2µm) *TRP1* | ([8](#_ENREF_8)) |
| *pGEN-rpb5-X* | ORI (2µm) *TRP1* | ([8](#_ENREF_8)) |
| *pGEN-Chi-X* | ORI (2µm) *TRP1* | ([8](#_ENREF_8)) |
| *pGEN-RPB7* | ORI (2µm) *TRP1* | ([8](#_ENREF_8)) |
| *pGEN-SPT15* | ORI (2µm) *TRP1* | This work |
| *pSCh202* | ORI (CEN) *URA3* | ([13](#_ENREF_13)) |
| *pSCh212* | ORI (CEN) *URA3* | ([13](#_ENREF_13)) |
| *pVV214* | ORI (2µm) *URA3* | ([14](#_ENREF_14)) |
| *pVV214-DST1* | ORI (2µm) *URA3* | Gift from M. Werner |
| *YCplac33* | ORI (CEN) *URA3* | ([15](#_ENREF_15)) |

**Table S3. Primers.**

| **Name** | | **Sequence** |
| --- | --- | --- |
| 18S-501 | | CATGGCCGTTCTTAGTTGGT |
| 18S-301 | | ATTGCCTCAAACTTCCATCG |
| 242 | | CTATTCGATGATGAAGATACCCCACC |
| 244 | | CAGTTGAAGTGAACTTGCG |
| 5S-501 | | GCGGCCATATCTACCAGAAA |
| 5S-301 | | CTGAGTTTCGCGTATGGTCA |
| ACT1-501 | | GCCTTCTACGTTTCCATCCA |
| ACT1-301 | | GGCCAAATCGATTCTCAAAA |
| ACT1P-501 | | TGAAACCAAACTCGCCTCT |
| ACT1P-301 | | GGTTTGAGTAGAAAGGGGAAGG |
| GAL1P-501 | | GCGAAGCGATGATTTTTGAT |
| GAL1P-301 | | TTCCTTTGCGCTAGAATTGAA |
| GAL1-501 | | TGGTGTTAACAATGGCGGTA |
| GAL1-301 | | GGGCGGTTTCAAACTTGTTA |
| GAL1-502 | | CATATGGTTCCCGTTTGACC |
| GAL1-302 | | ATAGACAGCTGCCCAATGCT |
| rDNAp-up | | AATTGAAGTTTTTCTCGGCGA |
| rDNAp-lo | | ATGAAGTACCTCCCAACTACTTTTCC |
| Intergenic Chr. V F | | TGTTCCTTTAAGAGGTGATGGTGAT |
| Intergenic Chr. V R | | GTGCGCAGTACTTGTGAAAACC |
| LACZ-501 | | ACTATCCCGACCGCCTTACT |
| LACZ-301 | | TAGCGGCTGATGTTGAACTG |
| LSM8-501 | | CCTTGTTTTGCTTGTTTTCC |
| LSM8-301 | | AGTCTTTCAAGGTGGCTGAC |
| LSM8-502 | | AATCAAAGTTGACGGCGAAT |
| LSM8-302 | | CTGCATCTATGAGGCCAACA |
| MBF1-501 | | TTAGGTAGCTGGAAAGGCGC |
| MBF1-301 | | GTTGTCACCCCTCGTATTGG |
| MBF1 -502 | | CGTGCCAGAACAGACAAGAA |
| MBF1-302 | | GCGAACCGATGTTGTTACCT |
| PHO5-501 | | AAACCACTTTTGCCAACTCG |
| PHO5-301 | | CGGATTCAGCTTCACTGACA |
| PMA1-P 503 | | AAAGGCCAAATATTGTATTATTTTCAA |
| PMA1-P 302 | | TTGGTGTTATAGGAAAGAAAGAGAAAA |
| PMA1-6 forw | | ATATTGTTACTGTCGTCCGTGTCTGGAT |
| PMA1-6 rev | | ATTAGGTTTCCTTTTCGTGTTGAGTAGA |
| PYK1-P-502 | | ACAAGACACCAATCAAAACAAA |
| PYK1-P-301 | | AGTCAGAACCAGCAACAACG |
| PYK1-4 forw | | CTATGGCTGAAACCGCTGTCATTG |
| PYK1-4 rev | | CAGCTCTTGGGCATCTGGTAAC |
| rDNA2 up | | AACAGTCTCATCGTGGGCA |
| rDNA2 lo | | TGAGAGGAGGTTACACTTGAAGAAT |
| rDNA5 up | | CAATAGCGTATATTAAAGTTGTTGCAGTT |
| rDNA5 lo | | AAAGTCCTGGTTCGCCAAGAG |
| RDN5S-up | | TCGCGTATGGTCACCCACTAC |
| RDN5S-lo | | GTTGCGGCCATATCTACCAGA |
| rDNA9-up | | CCAACCGGGATTGCCTTAGT |
| rDNA9-lo | | CACACGGGATTCTCACCCTCT |
| RPB5-502 | | GCCTTCCGTTGGTGTAAAGA |
| RPB5-302 | | CTTTGGAACCAATTCGTGGT |
| RPL25-501 | | TGGTTTTCCAAGTTTCCATGA |
| RPL25-301 | | CCAAAGCATCGTAGTCAGCA |
| RPL3-501 | | AAGAAGGCTCATTTGGCTGA |
| RPL3-301 | | AACACCTTCGAAACCGTGAC |
| RPL5-501 | | AGCTGACGACATTGATGCTG |
| RPL5-301 | | CACGAGCAGCTCTTTCTTCC |
| RPS15-502 | | GGTTCCGTCGTCGGTATCTA |
| RPS15-302 | | TGGGATGAAACGGGAAGTAG |
| RPS2-501 | | TGGTAAGTGTGGTTCCGTCA |
| RPS2-301 | | AATGGAGAAACTGGCAATGG |
| RPS3-501 | | TCTGGTCAACCAGTCAACGA |
| RPS3-301 | | TGGTTCAGCTTGAGCTTCAGT |
| SUP56 2 up | | TGACCCAAATATCACAAATAAGTGGTT |
| SUP56 2 down | | AGTTCACTGCGGTCAAGATATTTCT |
| TEF2-501 | | GCTTTGGTCAAGTTCGTTCC |
| TEF2-301 | | TAGCGGCCTTTTCAGTCTTG |
| TEF2p-501 | | TTCTTCTTCGACTATGCTGGAG |
| TEF2p-301 | | AGAAGGAGCGACACCAGAAA |
| 1-100 -3F (YLR1) | GATGTTTCCGATTAATGTTCTACTGTACAA | |
| 1-100 -66R (YLR1) | | GCTCCATAAGAAAGTCACTGCAAA |
| 1900-2000 -3F (YLR2) | | AGACAGAAGGAAATTTTACCAAGCG |
| 1900-2000 -63R (YLR2) | | AATCGAAAAAATCAGGTAGTTGCTG |
| 3800-4100 -191F (YLR3) | | GATATGCTTCAATCCGACAGAGAG |
| 3800-4100 -258R (YLR3) | | TCAACAGTTACCGATGGTATTAAAGG |
| 5900-6000 -14F (YLR4) | | AAATGGAACGAGGACGCAAG |
| 5900-6000 -84R (YLR4) | | GCAAATTCATTGGATAGGTTAGCA |
| 7600-7700 -21F (YLR5) | | GGCAAAGGAAAGATGAGATTGG |
| 7600-7700 -92R (YLR5) | | GTTGGACAATCTTAAAGTCGGGA |

**REFERENCES**

1. Garcia-Lopez, M.C., Miron-Garcia, M.C., Garrido-Godino, A.I., Mingorance, C. and Navarro, F. (2010) Overexpression of SNG1 causes 6-azauracil resistance in Saccharomyces cerevisiae. *Curr Genet*, **56**, 251-263.

2. Miron-Garcia, M.C., Garrido-Godino, A.I., Garcia-Molinero, V., Hernandez-Torres, F., Rodriguez-Navarro, S. and Navarro, F. (2013) The prefoldin bud27 mediates the assembly of the eukaryotic RNA polymerases in an rpb5-dependent manner. *PLoS Genet*, **9**, e1003297.

3. Navarro, F. and Thuriaux, P. (2000) In vivo misreading by tRNA overdose. *Rna*, **6**, 103-110.

4. Soutourina, J., Bordas-Le Floch, V., Gendrel, G., Flores, A., Ducrot, C., Dumay-Odelot, H., Soularue, P., Navarro, F., Cairns, B.R., Lefebvre, O. *et al.* (2006) Rsc4 connects the chromatin remodeler RSC to RNA polymerases. *Mol Cell Biol*, **26**, 4920-4933.

5. Harper, J.W., Adami, G.R., Wei, N., Keyomarsi, K. and Elledge, S.J. (1993) The p21 Cdk-interacting protein Cip1 is a potent inhibitor of G1 cyclin-dependent kinases. *Cell*, **75**, 805-816.

6. Van Mullem, V., Wery, M., Werner, M., Vandenhaute, J. and Thuriaux, P. (2002) The Rpb9 subunit of RNA polymerase II binds transcription factor TFIIE and interferes with the SAGA and elongator histone acetyltransferases. *J Biol Chem*, **277**, 10220-10225.

7. Flores, A., Briand, J.F., Gadal, O., Andrau, J.C., Rubbi, L., Van Mullem, V., Boschiero, C., Goussot, M., Marck, C., Carles, C. *et al.* (1999) A protein-protein interaction map of yeast RNA polymerase III. *Proc Natl Acad Sci U S A*, **96**, 7815-7820.

8. Zaros, C., Briand, J.F., Boulard, Y., Labarre-Mariotte, S., Garcia-Lopez, M.C., Thuriaux, P. and Navarro, F. (2007) Functional organization of the Rpb5 subunit shared by the three yeast RNA polymerases. *Nucleic Acids Res*, **35**, 634-647.

9. Gari, E., Piedrafita, L., Aldea, M. and Herrero, E. (1997) A set of vectors with a tetracycline-regulatable promoter system for modulated gene expression in Saccharomyces cerevisiae. *Yeast*, **13**, 837-848.

10. Bonneaud, N., Ozier-Kalogeropoulos, O., Li, G.Y., Labouesse, M., Minvielle-Sebastia, L. and Lacroute, F. (1991) A family of low and high copy replicative, integrative and single- stranded S. cerevisiae/E. coli shuttle vectors. *Yeast*, **7**, 609-615.

11. Rubbi, L., Labarre-Mariotte, S., Chedin, S. and Thuriaux, P. (1999) Functional characterization of ABC10alpha, an essential polypeptide shared by all three forms of eukaryotic DNA-dependent RNA polymerases. *J Biol Chem*, **274**, 31485-31492.

12. Wery, M., Shematorova, E., Van Driessche, B., Vandenhaute, J., Thuriaux, P. and Van Mullem, V. (2004) Members of the SAGA and Mediator complexes are partners of the transcription elongation factor TFIIS. *EMBO J*, **23**, 4232-4242.

13. Morillo-Huesca, M., Vanti, M. and Chavez, S. (2006) A simple in vivo assay for measuring the efficiency of gene length-dependent processes in yeast mRNA biogenesis. *FEBS J*, **273**, 756-769.

14. Van Mullem, V., Wery, M., De Bolle, X. and Vandenhaute, J. (2003) Construction of a set of Saccharomyces cerevisiae vectors designed for recombinational cloning. *Yeast*, **20**, 739-746.

15. Gietz, R.D. and Sugino, A. (1988) New yeast-Escherichia coli shuttle vectors constructed with in vitro mutagenized yeast genes lacking six-base pair restriction sites. *Gene*, **74**, 527-534.
