# Supplementary figures and images for: The yeast prefoldin-like URI-orthologue Bud27 associates with the RSC nucleosome remodeler and modulates transcription
Source: Nucleic Acids Res. 2014 Jul 31;42(15):9666–76. doi: 10.1093/nar/gku685 (PMC4150788; doi:10.1093/nar/gku685)

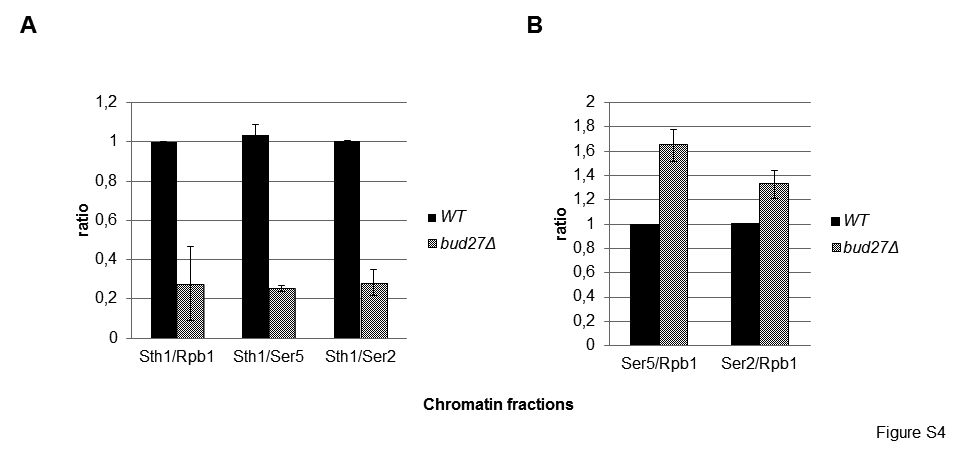

Supplement: SUPPLEMENTARY DATA [file supp_gku685_nar-03668-a-2013-File008.zip › FigS4 junio2014.tif]

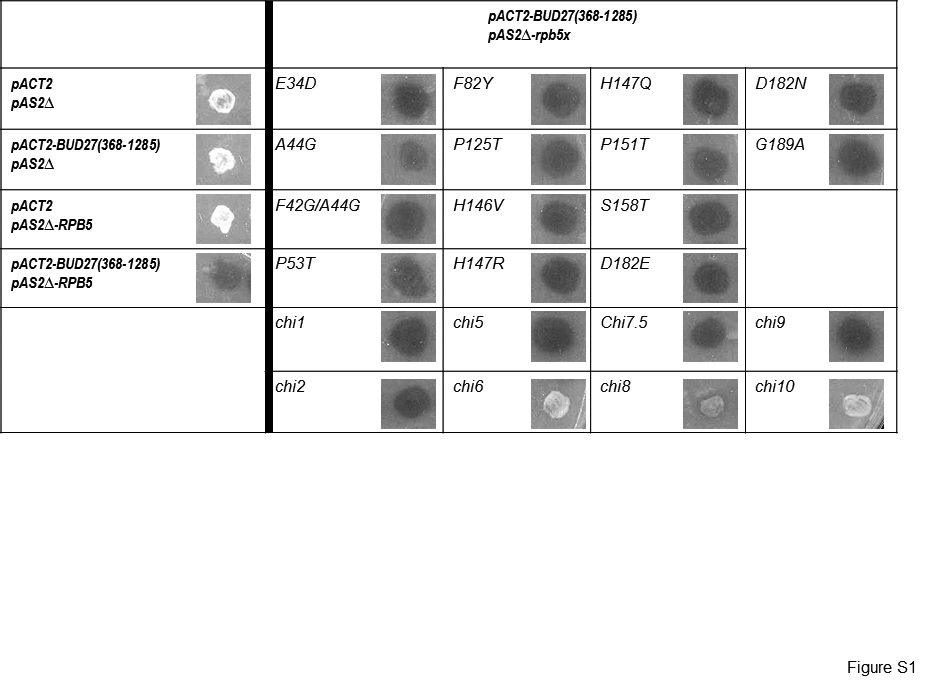

Supplement: SUPPLEMENTARY DATA [file supp_gku685_nar-03668-a-2013-File008.zip › FigS1 junio2014.tif]

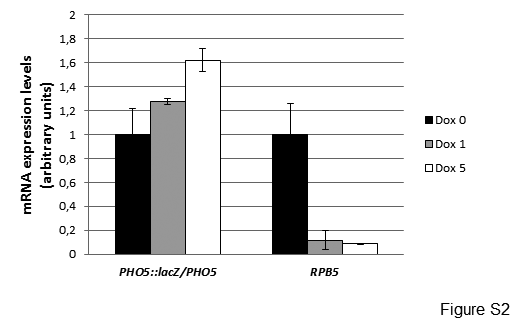

Supplement: SUPPLEMENTARY DATA [file supp_gku685_nar-03668-a-2013-File008.zip › FigS2 junio2014.tif]

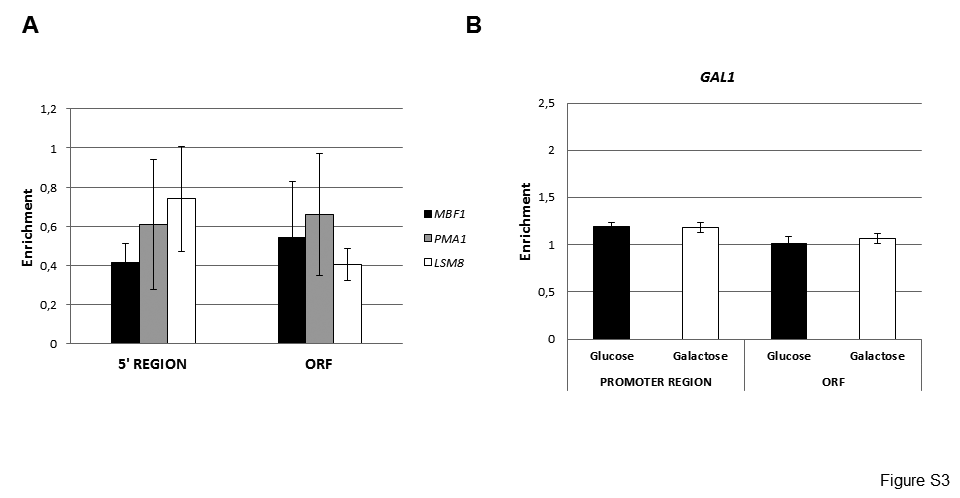

Supplement: SUPPLEMENTARY DATA [file supp_gku685_nar-03668-a-2013-File008.zip › FigS3 junio2014.tif]
